# Notes and News

**Published:** 1905-05-06

**Authors:** 


### Notes and News.

The site for the proposed Sanatorium for County Cork was the subject of lively discussion at the monthly meeting of the Cork Joint Hospital Board.

The honorary degree of LL.D. of Glasgow has been conferred upon Sir William Whitla, M.D. professor of Materia Medica and Therapeutics in Queen's College, Belfast.

A little while ago, Sir George White, of Bristol, gave £3,000 towards the Queen Victoria Memorial Hospital at Nice. Since then his brother, Mr. Samuel White, has offered to endow two beds in the proposed institution, and to place the nomination of patients to occupy them in the hands of the wife of the British Consul.

The Royal Institute of Public Health will hold a congress and exhibition on July 19 to 25, at the Polytechnic, Regent Street. The sections will include Preventive Medicine, Bacteriology and Chemistry, Tropical Hygiene, Veterinary Hygiene, Child Study and School Hygiene, etc. The municipal and educational authorities of the United Kingdom have been invited.

The annual meeting of the Invalid Children's Aid Association will be held, by kind permission of Mrs. Henry Tate, at Bolney House, Ennismore Gardens, S.W., on Wednesday afternoon, May 17, at 3 o'clock. Lord Midleton will preside, and Sir Henry Burdett, Professor Howard Marsh, and Mr. Pett Ridge will be among the speakers. Applications for tickets to be made to the Secretary, 69 Denison House, Vauxhall Bridge Road, Westminster, S.W.

The most important side of the dust question is undoubtedly the hygienic one, but reform is more likely to be effected for some other reason. The greatest inconvenience is experienced in the country, and therefore it is probable that the towns will have to wait until action is taken there. A well-known motorist has recently taken up the question, and he puts forward one or two preparations for the surface of roads, which are inexpensive and which have passed satisfactory tests.

Mr. Shepherd, of Rossend Castle, has given £10,000 to Gray's Hospital at Elgin, which is his native town.

The German Press at Prague is going to cooperate with the medical men to suppress quack medical advertisements.

Cerebro-meningitis has spread to Austria, and in the provinces of Galicia and Silesia the mortality has been very serious, the percentage amounting to about 50.

The Festival Banquet of the London School of Tropical Medicine, to take place on May 10th, is creating a great deal of interest. Mr. Chamberlain is to preside, and he is to be supported by a brilliant and important company.

The annual meeting of the Childhood Society will be held on Wednesday, May 17th, at 7 St. James's Square. Earl Egerton, of Tatton, will preside at 3 p.m., and Professor H. E. Armstrong will give an address on teaching "Common Sense."

Desford Hall has been opened as a convalescent home for Leicester and Leicestershire, and to serve as a memorial to Queen Victoria. Desford Hall has been adapted to the needs of 50 patients. The rooms which are large are arranged to contain five or six beds in each, a few one-bed wards being also provided. Ample space for recreation is provided within and without, and the establishment is placed under the care of a matron previously sister at the Leicester Infirmary.

It is obvious that the public are willing to give in charity in any direction when their sentiments are appealed to, and to give without inquiry or knowledge. The consequence is that any one may institute a collecting-box for some institution for an imaginary fund, and make quite a good living out of it. In the case of a man who was recently convicted at Hampstead, five to ten shillings a day was admittedly secured. The trick was detected by a police constable, and not by one of the victims, who apparently gave to a "free dinner fund" without inquiry.

It is desirable as a matter of public safety that the medical officer of health should be so secure in his position as to enable him to report and advise on sanitary conditions in his district without fear of the consequences from those to whom interference may not prove palatable. The medical officer of health properly safeguarded and equipped with energy, intelligence, and knowledge is one of the most valuable agents of civilisation. There are signs everywhere that he is recognising the importance of his mission and rising to it. Such an experience therefore as that of Dr. Francis Bond at Chipping Sodbury should be made impossible again. Dr. Bond, after 32 years' service, has been dismissed apparently for no other reason than because he declined to minister to an additional population of 4,600 without further remuneration. The Local Government Board, it appears, protested, but in vain.

